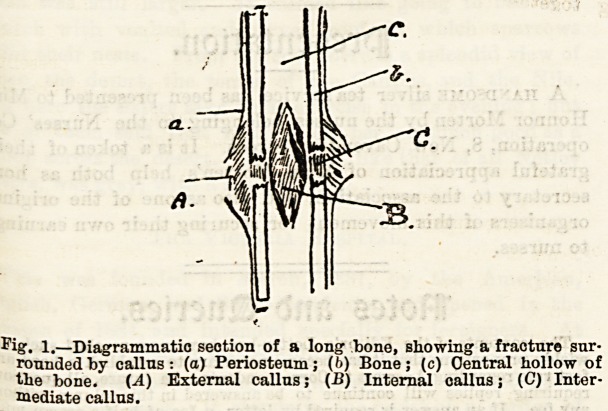# The Hospital Nursing Supplement

**Published:** 1895-03-09

**Authors:** 


*The Hospital*, March. 9, 1895. *Extra Supplement.*

### "The Hospital" Nursing Mirror.

Being the Extra Nursing Supplement of "The Hospital" Newspaper.
[Contributions for this Supplement should be addressed to the Editor, The Hospital, 428, Strand, London, W.C., and should have the word "Nursing" plainly written in left-hand top corner of the envelope.]

### News from the Nursing World.

### A QUEEN IN ISOLATION.

Those who have seen the Queen of Spain amongst her children, the little King being ever close by her side, can sympathise with the present compulsory isolation of this devoted royal mother. Her own convalescence happily now seems well established, but the attack of measles which the Queen has passed through still necessitates the banishment of her children to a different part of the palace. A telephone, however, enables the mother to hold personal converse with the young party, who eagerly anticipate rejoining the Queen.

### WOMEN LECTURERS.

At the third annual meeting of the Women Lecturers' Association, which was held last week, Mrs. Scharlieb, M.D., was one of the speakers. She considered the scheme most valuable as bridging over the abyss between the primary education of schools and the more advanced teaching of the University Extension lectures. In referring to the kind of teaching needed, Mrs. Scharlieb quoted the old phrase urging that it "may be understanded of the people." On the subject of nursing women, were undoubtedly the best teachers, for although men recognised and appreciated good nursing, they could not know how it was done so well as women trained to do it, just as it was commonly asserted that although men always knew when a woman was well dressed yet they would not be expected to explain how this result was arrived at. Mrs. Scharlieb also spoke of remunerative and congenial employment as the best "safety valve" for women's energies, and also as the best cure for certain ailments.

### PROBATIONERS' EXAMINATIONS AT LAMBETH.

Fifteen probationers and five nurses passed the examination held last month at Lambeth Infirmary, thereby securing their certificates. The written questions were ten in number, three hours being allowed to the candidates for stating their answers. The second part of the examination included a demonstration by each pupil of bandaging, poultice making, changing sheets, reading and charting temperatures, testing urine, and naming instruments. The examiners appointed by the Guardians were Dr. Bourns, of Westminster Hospital, and Dr. Webster, Medical Superintendent of St. George's Infirmary, Fulham, who must be congratulated on the thoroughness of their tests. Miss Griffiths and Miss Tilbury deserve congratulation on the results of the systematic instructions given, as the examiners highly commended many of the papers.

### MATERNITY WORK AT CLAPHAM.

Miss Helen Webb, M.D., presided at the annual meeting of the Clapham Maternity Hospital, which was held in the board room, Jeffreys Road, on March 1st. The general report, which was read by Miss Ritchie, the secretary, and adopted by the meeting, gave an account of the enlargement of the hospital premises, necessitated last spring by the rapid growth of the work. The balance-sheet showed a continued freedom from debt, the ordinary expenditure being £1,033 14s. 11d., and the ordinary income £1,121 0s. 10d. Only one death has occurred in the hospital since its opening in 1889. In the Clapham district 256 out-patients have been attended, and in Battersea 453, during the last year, whilst 302 in-patients were treated in the hospital. The whole of the maternity work is in charge of six qualified medical women, under whom obstetric pupils and nurses are trained. At the conclusion of the annual meeting the hospital was inspected by most of the visitors present.

### LIFE'S FAILURES.

The Countess of Warwick is likely to prove a powerful advocate of the introduction of trained night nurses into workhouse infirmaries. Speaking at a recent meeting of the Warwick Board of Guardians, with regard to this duty being left in the hands of paupers, she said, "To pick up nurses amongst life's failures could hardly result in securing many competent recruits for this work. As nine-tenths of the inmates were in the workhouse because they had no sense of responsibility to themselves, or those dependent upon them, it was hardly to be expected that their forgotten sense was likely to be developed in the infirmary; hence such people never could be trustworthy attendants upon the sick." The further remarks of Lady Warwick proved her appreciation of the responsibility resting on the guardians of the poor, and also showed the careful attention she had already given to the all-important necessity of adequate nursing being provided for the sick and infirm.

### NURSES AT YORK.

In the annual report of the York Home for Nurses the financial condition of the Charitable or Sick Poor Fund seems to be more satisfactory this year than that of the other branch of the work. In the previous report the Sick Poor Fund was burdened with a debt of £100, but this has now been paid off, £34 10s. 9d. being contributed by the working men of York in sums of less than half-a-crown. A sale of work in November and two organ recitals in York Minster resulted in substantial benefit to the fund. The York Home is under the management of the Sisterhood of the Holy Cross, and it appears that the demand for private nurses—namely, the paid branch of the work—has not been profitable during the last year. The nurses have not been sufficiently employed, and the doctor reports that he has had frequent occasion to visit them on account of illness. Three nurses have had to be suspended from active service on account of health, and this fact has drawn the attention of the council to the need for a provident fund, whilst it also demonstrates the wisdom of nurses joining the Royal National Pension Fund.

### QUEEN'S NURSES AT SOUTHAMPTON.

In the telegram in which Fleet-Surgeon Woods explained his inability to be present at the annual meeting of the Southampton branch of the Queen's Jubilee Institute, he referred to the great interest taken in the work by H.R.H. the Princess Henry of Battenberg. Surgeon-General Maclean, C.B., presided at the meeting, which was very well attended, and the report presented to the subscribers showed a satisfactory balance in hand.

### CAMBRIDGE.

It was announced at the annual meeting of the Cambridge District Nursing Association that the Guardians would repeat their subscription of £50. During last year 3,553 visits were paid to the destitute poor by the nurses, and 316 maternity cases were also undertaken. Systematic distribution of soup and beef-tea to the sick has been carried on during the year, different ladies providing the acceptable nourishment for the nurses to give away.

### NURSING AT BISHOP AUCKLAND.

The Bishop of Durham took the chair at the annual meeting of the Bishop Auckland District Nursing Association last week, and gave eloquent testimony to the value of the work done by it. Sir William Eden was elected president of the society, and Lady Eden president of the ladies' committee. It is hoped that increased interest in the work may be shown by working men in future, as their families benefit greatly by the skilled care of the district nurses.

### NIGHT NURSING NEEDED.

The Devonport Guardians had a report laid before them recently by their medical officer, to which they appear willing to attach due importance. There is urgent need for the adoption of many improvements in the care of the sick in the infirmary. At present from 150 to 170 poor creatures are left by day to the care of two untrained attendants, whilst paupers are in sole charge of the patients at night. In advising the employment of one trained nurse it was proposed that three untrained women should be engaged to assist her, one of whom should fill the office of night nurse. We hope shortly to learn that the necessity for the night nurse being fully trained and experienced has been acknowledged by those responsible for the humane treatment of the sick and aged by night as well as day.

### NURSING IN WALES.

At the annual meeting of the Bangor Nursing Institute a most satisfactory report was laid before the meeting regarding the year's work. The Mayor, Dr. Langford Jones, presided at the meeting, which was largely attended, and in addition to the staff of private nurses, he remarked that Queen's nurses were engaged in district work amongst the poor of Bangor, Carnarvon, Bethesda, Menai Bridge, Conway, and Beaumaris.

### DISTRICT NURSING IN IRELAND.

The encouragement given to trained nursing for the poor in Ireland appears to be increasing, and the formation of a new district nursing association is under discussion at Mitchelstown, a committee being appointed to go into the matter. Miss Dunn, general superintendent of the Irish branch of the Queen's Jubilee Institute, is to be invited at an early date to speak at a general meeting, when the question of affiliation with the institute will be brought forward.

### ST. PATRICK'S NURSES' HOME.

The annual meeting of the friends and supporters of St. Patrick's Nurses' Home (affiliated with the Queen's Jubilee Institute) was held on Tuesday, 19th ult., in the Gregg Memorial Hall. Lord Plunkett, Archbishop of Dublin, presided. The institution maintains at the present time eleven nurses, working amongst the poor in their own homes, 28,000 visits having been paid by them during the past twelve months. Seven ladies have been trained for district nursing in connection with the Queen's Jubilee Institute, five of whom are now at work in various country districts in Ireland; one remains on the staff as a "St. Patrick's" nurse, while a Dutch lady, Miss Kruyses, has returned to Holland to become one of the pioneers of district nursing in that country. Dr. FitzGibbon spoke from experience as medical officer to the General Post Office as to the good work done by the Jubilee nurses amongst the sorters, telegraph clerks, postmen, messenger boys, &c., who suffered much from illness during the Christmas season owing to overwork. The home has sustained a great loss in the death of Miss Thompson, late hon. secretary, in memory of whom it is proposed to establish a small library for the use of the nurses, as well as to erect a window in the Lady Chapel of St. Patrick's Cathedral. The financial report is satisfactory, £208 remaining in hand after payment of all annual expenses.

### FAITHFUL TO DUTY.

A fire broke out last month at the Deaconesses' Hospital, Cleveland, U.S.A., and the danger was not realised until it was too late to rescue three patients and a nurse, who all unhappily perished. The young nurse, Minnie Baumer, could have escaped along the roof of the verandah, but she bravely refused to desert a helpless patient.

### A NEW YORK CLUB.

The conditions of membership in the New York Trained Nurses' Club are fulfilled by candidates with two years' training in "a respectable training school of fifty beds or more." The present club owes its origin to Miss Kate Teachman, who undertook the whole trouble of organising and financing it. There are now a large number of members who live at the club between private cases, and have inaugurated a Mutual Benefit Association for Sick Nurses.

### SHORT ITEMS.

The Smethwick and District Nursing Association had a good attendance at the annual meeting.—At Twickenham last month the second annual meeting took place of the Holy Trinity District Nursing Association, which is affiliated with the Queen's Jubilee Institute.—*Nursing Notes* for March contains some very good articles, notably "Law Notes for Nurses," by a Barrister.—The London Young Women's Christian Association has opened a large home for students, governesses, &c., at Kent House, 89 and 91, Great Portland Street, London, W., where there are cubicles to accommodate seventy or eighty boarders.—Two concerts given at Exeter last month in aid of the Heavitree Rural Nurses' Association were well attended.—The Bolton Guardians are discussing the question of a Home for their nursing staff.—Miss de Pledge, matron of Chelsea Infirmary, is giving a course of lectures on nursing (under the auspices of the National Health Society), at Queen's House, Chelsea, by kind permission of Mrs. Haweis.

### Elementary Anatomy and Surgery for Nurses.

By W. McAdam Eccles, M.B., M.S., F.R.C.S., Lecturer to Nurses, West London Hospital, &c.

### VIII.—FRACTURES IN GENERAL.

A fracture has been defined as the sudden solution of the continuity of a bone, or to put it more simply the sudden breaking of a bone into two or more pieces. Such a result may be brought about in either of two ways—(1) Direct violence, as occurs when a bone is broken by a blow directly applied to it; (2) indirect violence, as when a fall on the palm of the hand causes a fracture of the clavicle. A bone broken by muscular action might also be said to be fractured by indirect violence.

A fracture is called simple when there is no communication by a wound of the soft parts between it and the external air. It is designated compound when the fracture does communicate with the surrounding air. The distinction between these two varieties is of the utmost importance. Moreover, a bone may be broken into more than two pieces at the seat of fracture, this is known as a comminated fracture.

If in addition to the break of the bone itself there is grave injury to the soft parts, such as the rupture of a large blood-vessel, a complicated fracture has occurred. A complete fracture is one where the bone is broken into two separate pieces, an incomplete or "green-stick" fracture is

Fig. 1.—Diagrammatic section of a long bone, showing a fracture surrounded by callus: (*a*) Periosteum; (*b*) Bone; (*c*) Central hollow of the bone. (*A*) External callus; (*B*) Internal callus; (*C*) Intermediate callus.

one where the bone is only partially broken across. This happens most frequently in the softer bones of children.

The chief symptoms or signs by which a fracture may be diagnosed should be known by a nurse; they are—(1) Displacement. This is very often most obvious. There is an alteration in the shape, length, or direction of a limb, or other part. The "setting" of a fracture is the correction of such deformity. (2) Crepitus. This is the rough grating of the broken ends of the bones when they come in contact with each other. This sign is absent in some fractures, such as green-stick, and those in which the fractured ends become interlocked or impacted. (3) Pain and swelling about the seat of injury. (4) Abnormal mobility because of the breaking of the bone into two pieces. (5) Loss of the normal function of the part.

The mending or union of a broken bone is accomplished by Nature in practically the same way as she heals wounds of the soft tissues. Blood is poured out into the tissues almost immediately after the fracture, and then there follows plastic inflammation, whereby the broken ends are surrounded with a mass of inflammatory exudation, which is known as soft callus. In a long bone this is formed outside the bone, in the central canal of the bone, and also between the actual fragments themselves. (See Fig. 1.) This soft callus is converted into fibrous tissue, which in its turn becomes ossified or changed into bony tissue called hard callus, the excess of which is removed later. In six weeks most fractures are pretty firmly united, but it will be many months before the part returns to its original condition. About a compound fracture suppuration may occur and healing be much delayed, or never properly take place.

A nurse who has to deal with a case of fracture in a hospital before the arrival of the surgeon should observe the following directions—(1) See that all clothing about the fracture is cut off, not pulled off. If the latter method be used there may be great danger in rendering a simple fracture compound. (2) Take care that the fractured limb or part is moved as little as possible, and that in the gentlest manner. (3) If the fracture be compound cover the wound with some lint or wool wrung out of an antiseptic solution. (4) Prepare the required splints and other apparatus requisite for the treatment of the special fracture. (5) Get ready the bed (if the fracture be of one of the lower limbs) by placing a firm hard mattress upon it.

In the general treatment of fracture these points should be remembered—(*a*) The fragments need to be reduced, that is, brought into apposition so as to restore the normal shape of the bone. (*b*) Having been reduced, they require to be kept so, and at the same time maintained at rest. This is generally effected by means of splints. (*c*) In a compound fracture the wound must be treated antiseptically. After a fracture has been "set" it is highly important to see that none of the bandages become tight from the swelling of the part which is likely to occur, as this may lead to serious interference with the circulation.

### Burdett's Official Nursing Directory.

In reply to numerous inquiries, we beg to state that any nurse who desires to have the great advantage of appearing in this official directory can obtain a form and all necessary particulars on sending a stamped and directed envelope to the Editor B. O. N. Directory, 428, Strand, W.C. As we pointed out last week, every nurse ought to make sure that whatever list or register she may be on her name shall also appear in "Burdett's Official Nursing Directory." Its success is already assured, and the medical profession and the public, as well as the nurses, are manifesting an increasing interest in its progress.

### Where to Go.

Queen's Hall, Langham Place.—Organ recitals every Sunday at half-past three. Admission free.

Queen's Hall, Langham Place.—A grand evening concert will be given by the Strolling Players' Amateur Orchestral Society on March 13th, in aid of the Metropolitan Hospital, Kingsland Road. H.R.H. the Duchess of Teck has kindly promised to be present.

Mansion House.—On Tuesday, March 12th, the annual meeting of the East London Nursing Society will be held at three p.m.

### Wants and Workers.

[The attention of correspondents is directed to the fact that "Helps in Sickness and to Health" (Scientific Press, 428, Strand) will enable them promptly to find the most suitable accommodation for difficult or special cases.]

Emily Baker, on whose behalf the Matron of Lyme Regis Cottage Hospital appealed to our readers last week, is a respectable, industrious woman, who formerly made a living by the aid of a knitting machine. For several months she has been an inmate of the cottage hospital, incapacitated by spinal disease. After a wearisome illness she is now so far recovered that provision for her future becomes a pressing need. Subscriptions to maintain her for a time will be thankfully received and acknowledged by Miss Eger, Matron, Cottage Hospital; or Mr. B. W. Hillman, Lyme Regis.

### Notes from St. Helena.

By Rose A. Blennerhassett, Lady Superintendent of the Civil Hospital.

### V.—NATURAL BEAUTY OF THE ISLAND.

The indigenous plants are few, and all of them white, owing to the absence of fertilising insects. At one time the island was thickly wooded, and dense forests covered hills where now prickly pears alone flourish, and in those days there appear to have been numbers of snails of a kind now extinct in the island. A very interesting visitor from New Zealand, who stayed here *en route* for England, collected some of the fossil shells of these snails, which are now only found in one place. This doctor had formerly been colonial surgeon here, and when quite a boy had known the famous Dr. Barry, who was then the senior military doctor at St. Helena. No one, it appears, had ever had the faintest suspicion that the distinguished army surgeon, prancing about on a beautiful white horse, was a woman! "The only thing," said our visitor, "which might have led to such a discovery was the fact that Dr. Barry was extraordinarily quarrelsome for a man." There were other and more creditable circumstances which led to Dr. Barry being considered eccentric. In those days of "three bottle men" the army surgeon was an almost total abstainer, and was in all ways so remarkably abstemious, that he (or she) was credited with having adopted the religion of Brahma when stationed in India. I wish I could give an idea of the strange effect of European and tropical trees and flowers all growing together in picturesque confusion. Datura trees, with great trumpet-shaped flowers that emit an intense enervating perfume as soon as the sun sets, rub shoulders with sturdy oak; homely periwinkles blossom in the shadow of gardenia bushes. You see an honest English holly bush with a brilliant cardinal bird perched on it, and a tall bamboo waving its graceful branches in the background. Every patch of moist shady ground is now white with arum lilies in full bloom, the slopes of grassy hillsides are literally ablaze with the glory of blossoming gorse. Anything and everything thrives at St. Helena.

### St. Helena Coffee.

The Governor grows his own coffee. It is so excellent that those who taste it always wonder that the island is not the centre of a great coffee industry. But the St. Helenians have for ages been accustomed to trust little to their own industry. They depend chiefly on the money spent by the ships which touch here. Formerly, when the ships could be counted by hundreds, the islanders were very prosperous, now they are poor, and the young people emigrate largely to the Cape. As yet, however, poverty has not developed energy. They subsist on fish and coarse rice, which is largely imported, drinking a poisonous beverage supposed to be tea. The country people live to an extreme old age, and are strong and active almost to the last. A woman of 85 will tramp up and down hill, doing 12 or 14 miles a day without thinking twice of it.

### The Island as a Health Resort.

We are assured that St. Helena would make a splendid health resort, and people often wonder that no one has opened a sanitorium at Longwood. The climate is said to work wonders for bronchial trouble, and to be very beneficial in diseases of the kidneys and intestines. The society is at present small, consisting of some eight or ten families, chiefly those of officials. It is a simple, good natured, and hospitable society, meeting every two or three days for tennis, croquet, or archery.

Once a year St. Helena holds a sort of carnival. The arrival of the flagship initiates a week of wild dissipation. Dinners, dances, and picnics succeed each other with bewildering rapidity, and perhaps the most enjoyable of these festivities is the dance on board, which the admiral usually gives.

Events are dated from these visits. Before the flagship arrives St. Helena pines for it, orders new frocks, and studies cookery books. For some months afterwards it remains a source of unfailing conversation and interested discussion. We arrived just in time to enjoy the carnival, which gave us the somewhat mistaken impression that life at St. Helena was one whirl of excitement. In reality existence here is in most ways similar to that of a quiet country neighbourhood in England—a resemblance increased by the constant rain. The natives, too, all speak English. They are of mixed blood, the descendants of slaves and servants of the East India Company, and of all shades of colour. They are gentle, pretty-mannered, inoffensive people, crime being almost unknown on the island.

It was with much reluctance that our decision to resign work here was arrived at, but the experiences in Mashonaland were so injurious to health that we find nursing must be given up altogether. It is scarcely necessary to say to nurses how deep is our regret at being obliged to leave the ranks of a profession regarded by us with pride and reverence.

### Presentation.

A handsome silver tea service has been presented to Miss Honnor Morten by the nurses belonging to the Nurses' Co-operation, 8, New Cavendish Street. It is a token of their grateful appreciation of Misa Morten's help both as hon. secretary to the association, and also as one of the original organisers of this movement for securing their own earnings to nurses.

### Notes and Queries.

The contents of the Editor's Letter-box have now reached such unwieldy proportions that it has become necessary to establish a hard and fast rule regarding Answers to Correspondents. In future, all questions requiring replies will continue to be answered in this column without any fee. If an answer is required by letter, a fee of half-a-crown must be enclosed with the note containing the enquiry. We are always pleased to help our numerous correspondents to the fullest extent, and we can trust them to sympathise in the overwhelming amount of writing which makes the new rules a necessity. Every communication must be accompanied by the writer's name and address, otherwise it will receive no attention.

### Queries.

(90) *Cottage Hospital.*—I should be glad to know of suitable preliminary studies to fit me for cottage hospital work.—*Edith.*

(91) *English Nurses in Paris.*—Is there a work published which gives an account of the Paris hospitals, as I want the name of the hospitals in Paris where English physicians are on the medical staff, and where English fully-qualified nurses are received?—*S.W.*

(92) *Attendant.*—I am a trained hospital attendant and handy man, and require work.—*E. J. H.*

(93) *Health Lectures.*—Can you oblige me with the address of the National Health Society?—*Matron.*

(94) *Training.*—Where can I obtain post as probationer at a general London hospital without a premium, at the age of 20?—*N.B.*

(95) *Hospital Furniture.*—I shall be grateful if you will give me the name of the best firm for supplying hospital furniture?—*Matron.*

### Answers.

(90) *Cottage Hospital (Edith).*—In addition to having full general hospital training, a nurse taking charge of a cottage hospital should possess a thorough knowledge of every branch of household management. "Edith's" studies should, therefore, be directed to making her a good housekeeper and needlewoman, as well as an accomplished nurse. The experience gained in a cottage hospital is varied and valuable, but can only be duly appreciated by those qualified for it. If you want to study books you should order "Burdett's Cottage Hospitals," at 428, Strand. Works on general nursing, domestic economy, and invalid cooking, will also be useful.

(91) *English Nurses in Paris (S.W.).*—If you refer to page 404 of "Burdett's Hospital Annual for 1894," you will find the information you want.

(92) *Attendant (E. J. H.).*—We can only advise you to study advertisements or to advertise yourself.

(93) *Health Lectures (Matron).*—N.H S., 53 Berners Street, London.

(94) *Training (N.B)*—If you look back you will see this question has often been answered in this column. You are too young to begin training. Only some children's and fever hospitals take probationers of 20.

### Nursing in Cairo.

By Mrs. Brewer.

### The Military Hospital in the Citadel.

This is in some ways similar to other military hospitals inasmuch as it is nursed by Netley sisters, and possesses those characteristics of order and discipline which mark all institutions connected with the army; but here the similarity ends. Where else could be found one to compare with it in beauty of decoration, having been once the Palace of Saladin? We approached by a beautifully-kept garden, up the chief marble staircase, into a cruciform hall elaborately carved containing, on the day of our visit, 120 patients. The sisters are not allowed to go near infectious cases, which are nursed by orderlies in an isolated house.

For the wives and children of the soldiers a women's hospital is attached to the barracks, but they are usually nursed in their own quarters.

The marble floors and staircases of this magnificent building suggest a palace rather than a hospital, and the chapel for the staff, formerly the bath, is exceptionally beautiful.

From the large hall two corridors lead to the sisters' quarters, the rooms of the head sister being enormous; the sitting-room required four lamps to light it, but the bedroom was still larger. It seemed like going to bed in a church with vaulted and carved roof in which sparrows build their nests. From the roof there is a splendid view of Cairo, the desert, the tombs of the Caliphs, and the Nile, and here the sisters take their exercise in the evening; but one of them said that nothing did her so much good as a gallop across the desert. The dress, like that of all Netley sisters, was grey, with a scarlet cape.

### The Victoria Hospital.

This was founded in March, 1881, by the American, English, German, and Swiss residents, and opened in the autumn of 1884, and intended specially for foreigners. At the time of our visit it was simply a cottage hospital, but has since been enlarged and improved, and has an isolated house for infectious cases. It stands in the midst of a garden of palms, and is presided over by six deaconesses from Kaiserworth, and is frequently called the Deaconesses' Hospital. It is bound to take German, Swiss, English, and American patients, but Greeks and Arabs are also received.

There are three classes of payment—first-class, 50 piastres or 10s. a day; second-class, 25 piastres or 5s. a day, and the third-class 12 piastres. The sick seem well cared for, the food good and well prepared, and the rooms clean and comfortable.

The sisters, kind, sympathetic, and efficient, seem rather over-worked. The head one was for eleven years in a hospital in Jerusalem before coming here.

The out-patients are received in a building set apart for them, and are mostly suffering from ophthalmia.

### The Dispensary for Jews.

This is not situate in the Jews' quarter, but on the other side of the Mousky, and is the outcome of the Rev. Nasar Odeh's work. It is open daily from half-past two for as long as it is required. There is no Jews' hospital at present, and they have a great objection to going into the native Government hospital, and often remain at home ill to the disadvantage of the whole Jewish quarter.

The dispensary has a small waiting-room attached, and a book wherein names and diseases are entered. Various doctors give their aid gratuitously, and lady visitors keep the books when many patients are waiting to be attended to.

### Everybody's Opinion.

### THE UNAUTHORISED ADVERTISEMENT NUISANCE.

"The Matron of a Small Hospital" writes: I also have had to complain of advertisements inserted without authority or payment in the *Nursing Record*. Can nothing be done to put a stop to the nuisance?

### THE MASSAGE QUESTION.

Mr. E. Luke Freer writes: I have to thank you for the insertion of my letter on this subject, and for your foot-note enlightening me as to the existenee of another association besides that mentioned. If this is being carried on with the co-operation of the medical profession, it will be welcomed by both doctors and operators; it is, however, strange that I should not have heard of its existence, as I made repeated inquiries of operators, both here and in London, before I formulated my suggestions which first appeared in the medical journals twelve months ago.

### LONDON OBSTETRICAL SOCIETY.

"A Medical Correspondent" writes: In The Hospital of March 2nd, page 382, you state that Dr. W. S. Playfair has published the exact wording of the so-called certificate granted by the Obstetrical Society. If you will look at the original you will probably be as astonished as I was when I read Dr. Playfair's letter, that he has left out the most important clause. I know how desirous you are to be accurate, and with such a feeling I call your attention to this sad error.

[In accordance with our correspondent's suggestion we have read the original certificate, which runs as follows:—

"Obstetrical Society of London.

"We hereby certify that has passed, to our satisfaction, the examination instituted by the Obstetrical Society of London, and that she is, in our opinion, a skilled midwife, competent to attend natural labour." (Here follow the doctors' signatures).

"I undertake to abide by all the rules and regulations of the Obstetrical Society with regard to the duties and conduct of midwives, and to submit to the jurisdiction of its council in the decision of all matters relating to my conduct as a midwife. I further agree that in case I shall hereafter be convicted of any criminal offence, or be guilty of any act or conduct which, in the opinion of the council, renders me unfit or unworthy to hold this diploma, the same may be forfeited by resolution of the council, in which case I will, on receiving notice in writing of such resolution, to be served either personally or by leaving the same at my then present or last-known place of abode in the United Kingdom, forthwith give up such diploma to the president or to one of the secretaries for the time being of the society, and I agree that my name may be removed from the registry of midwives kept by the society, and I promise thenceforward to desist from the use of any designation or title implying possession of such diploma." (Signed by midwife.)

What our correspondent speaks of as "the most important clause," is no part of the statement signed by the officers of the Obstetrical Society, but is a mere undertaking signed by the midwife, and in no way invalidates our suggestion that it is a misuse of language to speak of the certificate itself as a colourable imitation of a "license to practise medicine, surgery, and midwifery."—Ed. *T. H.*]

### National Health Society.

The course of lectures on Domestic and Personal Hygiene, given at 53, Berners Street, by Dr. A. T. Schofield on Tuesdays and Fridays at 3 p.m., concludes on March 15th.

Lectures on Domestic Economy are given by Mrs. Dickson on Thursday afternoons at 3 p.m. at the same address.

### The Book World for Women and Nurses.

[We invite Correspondence, Criticism, Enquiries, and Notes on Books likely to interest Women and Nurses. Address, Editor, The Hospital (Nurses' Book World), 428, Strand, W.C.]

Good Reading about Many Books, mostly by their Authors. (T. Fisher Unwin, London.)

Mr. Fisher Unwin has carried out a most happy idea in publishing a collection of the why and wherefore many authors wrote their books. This opportunity has often been longed for by an author after his work has been launched on the sea of public opinion. Misunderstanding, even fibs, respecting their books have to be borne in silence by authors who have not influence with the press. Under Mr. Fisher Unwin's system the author gets his fair hearing and opportunity. From the reader's point of view the innovation is equally happy. When a work has succeeded in attracting attention the public feel the writer is a matter of general property, and his personality at once becomes an interest. In "good reading" the various writers have a talk with the public, and the conversation is varied and entertaining. John Oliver Hobbs, S. R Crockett, F. Edmund Garrett, Grant Allen, and many others are amongst those who have joined the interesting conversazione. As a preface to the conversation, a portrait of the author is given, and but one alteration is needed in our view to enhance the value of the collection. This is, that each article should be separate, and not printed on the back of the page of another, so that these words about themselves might find a place within the original book referred to. As we hope the present volume is only the commencement of a series, we trust Mr. Fisher Unwin will enhance the interest of the future collections thus.

Domestic Hygiene. By Thomas Dutton, M.D. (London: Henry Kimpton and Hirschfield Bros. 1894.)

This small volume is composed of short chapters on matters of domestic hygiene, each of which, Dr. Dutton tells us in the preface, was originally written for special journals. This accounts for what is a serious defect in an otherwise useful work, the number and variety of the subjects compressed into its 199 small pages of large print. We do not think even "the rudiments of hygiene" can bear such sketchy treatment, and half the quantity of matter, more fully dealt with, would have been of far more value to the "general reader" for whose enlightenment the manual is intended. This brevity of treatment naturally leads to somewhat vague suggestions, such as the following in regard to the purification of the atmosphere: "To purify air from germs pass it through red-hot tubes and so destroy them; if (*sic*) to intercept them pass the air through cotton wool," which, unfollowed by any directions as to the carrying out of the experiments, will be of little practical use to the ordinary reader. The lay public have been provided with so many "useful manuals" of information on matters sanitary and hygienic that he is a brave man who ventures to add another to their number unless it has some very special recommendation. We do not fancy "Domestic Hygiene" can fairly be placed in this latter class.

The Mother's Help and Guide to the Domestic Management of Her Children. By P. Murray Braidwood, M.D., F.R.C.S. (Scientific Press: 428, Strand.)

The experience of other writers is added to the personal observations of Dr. Braidwood in this volume, to which he appends a list of eight authors he has quoted from. His own remarks on the ventilation of children's nurseries are excellent. We read, "When children awake in the morning not refreshed by their night's rest, and are indisposed to get out of bed, it is often supposed that their health is affected, whereas this condition results merely from their breathing during the night the air of a close bed-room." Many hints useful to mothers and others who have the care of infants are given by Dr. Braidwood, not the least valuable being the following: "Every mother should bear in mind that her duty is to observe and note symptoms, but the doctor should be asked to interpret them." Our own experience of children does not, however, lead us to share the opinion that "healthy children, as a rule," don't care for sweets!

### For Reading to the Sick.

### TRIAL OF FAITH.

**Motto.**

I will do or suffer what I ought.—*George Herbert.*

**Verses.**

When God afflicts thee, think He hews a rugged stone,
Which must be shaped, or else aside as useless thrown!
—*Trench.*

Faith alone can interpret Life — and the
Heart that aches and bleeds with the stigma
Of pain, alone bears the likeness of Christ,
And can comprehend its dark enigma.
—*Longfellow.*

Men as Men
Can reach no higher than the Son of God,
The Perfect Head and pattern of mankind.
The time is short, and this sufficeth us
To live and die by; and in Him again
We see the same first starry attribute,
"Perfect through suffering," our salvation's seal,
Set in the front of His humanity . . . .
While we suffer, let us set our souls
To suffer perfectly: since this alone—
The suffering — which is this world's special grace,
May here be perfected and left behind.
—*H. Hamilton King.*

Why should I then my pains decline,
Inflicted by pure love Divine?
Let them run out their destined course,
And spend upon me all their force:
Short pains can never grievous be
Which work a blest eternity! —*Bishop Ken.*

**Reading.**

Stars shine brightest in the darkest nights; torches are better for beating; grapes come not to the proof till they come to the press; spices smell best when bruised; young trees root the fastest for shaking; gold looks brighter for scouring; juniper smells sweetest in the fire; the palm-tree proves the better for pressing; camomile, the more you tread it, the more you spread it. Such is the condition of all God's children; they are then most triumphant, when most tempted; most glorious when most afflicted; most in the favour of God when least in man's and least in their own; as their conflicts, so their conquests; as their tribulations, so their triumphs; true salamanders, that live best in the furnace of persecution; so that heavy afflictions are the best benefactors to heavenly affections; and where afflictions hang heaviest, corruptions hang loosest; and grace, that is hid in nature, as sweet water in rose leaves, is then the most fragrant, when the fire of affliction is put under to distil it out. *C. H. V. Bogatozky, "Golden Treasury."*

A child rejects with abhorrence the medicine which will heal its pain; but experience of its known virtue makes the grown man to long for, and to value it. So the new-born soul shrinks from, and struggles against the trials of life, while the more mature Christian quaffs to the dregs with faith those bitter tonics which refresh and invigorate his soul.
—"*Heart Musings.*"

## Figures and Tables

**Fig. 1. f1:**